# Pancreatitis after percutaneous ethanol injection into HCC: a minireview of the literature

**DOI:** 10.1186/1756-9966-27-28

**Published:** 2008-08-14

**Authors:** Enrico M Zardi, Francesco Di Matteo, Daniele Santini, Valentina Uwechie, Pierfilippo Crucitti, Massimiliano Carassiti, Antonio Picardi, Eleonora Perrella, Marco Caricato, Giuseppe Tonini, Roberto Coppola, Antonella Afeltra

**Affiliations:** 1 Department of Clinical Medicine, "Campus Bio-Medico" University, Rome, Italy; 2 Department of Digestive Diseases, GI Endoscopy Unit, " Campus Bio-Medico" University, Rome, Italy; 3 Medical Oncology, "Campus Bio-Medico" University, Rome, Italy; 4 Department of Infectious and Tropical Diseases, University "La Sapienza", Rome, Italy; 5 Department of General Surgery, "Campus Bio-Medico" University, Rome, Italy; 6 Department of Anesthesia, "Campus Bio-Medico" University, Rome, Italy; 7 Department of Pathology, "Campus Bio-Medico" University, Rome, Italy

## Abstract

Deaths after percutaneous ethanol injection (PEI) into hepatocellular carcinoma (HCC) may occur within a few hours to a few days following the procedure because of hemoperitoneum and haemorrhage from oesophageal varices or hepatic insufficiency. Pancreatitis has been recently reported as a rare lethal complication of intra-arterial PEI, another modality for treating HCCs. In this minireview, we analyze the literature concerning the development of acute pancreatitis after PEI. Pathogenesis of pancreatitis from opioids and ethanol is also addressed. Treatment with opioids to reduce the patient's abdominal pain after PEI in combination with the PEI itself may lead to direct toxic effects, thus favouring the development of pancreatitis.

## Review

Percutaneous ethanol injection (PEI) is a widely used procedure for the treatment of hepatocellular carcinoma (HCC), and may be performed via conventional, "one shot" or intra-arterial modalities.

While conventional PEI is performed under localized anaesthesia and the amount of ethanol injected into the HCC generally does not exceed 10 ml/session, "one shot" PEI is performed under general anaesthesia and the amount of administered ethanol is higher, ranging from 20 to 60 ml/session. Intra-arterial PEI is also performed under general anaesthesia, but ethanol (up to 50 ml) is directly injected, through a percutaneous route, into the artery that supplies the HCC after visualizing and puncturing this artery by using colour Doppler and B-mode ultrasound guidance. Interestingly, as demonstrated in a cell culture experimental study on malignant and liver cell lines, the cytotoxic effect of ethanol is dependent upon both its concentration and the exposure time [1].

Since at present, the concentration of ethanol is standardized to 95% and the exposure time of the HCC is considered to be practically identical in the two PEI procedures, our opinion is that the development of complications may only depend on the high total dosage of ethanol injected and the patient's clinical conditions. However, according to some authors, no difference in complications (pain and fever excluded) has been reported when using larger doses of ethanol [2].

The most frequently reported complication of these three PEI modalities is abdominal pain that may be observed in up to 48% of cases [3].

If pain is not tolerated, especially when the doses of injected ethanol are high, the administration of nonopioid or mild opioid analgesics may be required [3]. Since cases of acute pancreatitis after opioid administration have been reported [4-17], we believe that more attention must be given when such drugs are administered. In fact, it is ascertained that there is a close temporal relationship (ranging from 1 to 3 hours) between opioid administration and the development of pancreatitis [7,18].

A number of physiopathological studies have elucidated the mechanism through which opioids may induce pancreatitis. These studies most often implicate direct constriction of the sphincter of Oddi [18]; in fact, it has been demonstrated that intravenous morphine increases the intrabiliary pressure by enhancing sphincter of Oddi pressure [14]. It has also been shown that, after biliary sphincterotomy, pancreatitis may occur due to the sphincter spasm [7]. Taking into consideration that sphincter of Oddi dysfunction, a clinical syndrome due to a dyskinesia resulting from a functional alteration of sphincter motility or to stenosis, may occur at any age [19], our opinion is that it should be excluded before giving opioids after PEI in patients with HCC. This caution is very important considering that in patients with idiopathic recurrent pancreatitis, manometric evidence of sphincter of Oddi dysfunction was found to vary between 39 and 90% [20]. Furthermore, most cirrhotic patients with HCC suffer from cholelithiasis, and acute pancreatitis has been reported to occur in association with secondary sphincter of Oddi dysfunction, which is related to biliary calculi in 90% of cases [21]. According to some authors, since cholecistectomy would seem to favour the development of acute pancreatitis after ingestion of therapeutic doses of opioids [7], we believe that pain management with opioids after PEI treatment in cholecistectomized cirrhotics with HCC should be performed with great caution.

Furthermore, interesting animal studies have demonstrated that ethanol may have direct effects on the pancreas, such as microcirculatory changes and direct toxic damage to the pancreatic acini [22-24]. Moreover, the mechanism of ethanol-induced pancreatitis has been well-studied in an interesting animal model in which it was demonstrated that sphincter of Oddi dysfunction was implicated in several forms of acute and chronic pancreatitis [25]. In fact, according to the authors, since trans-sphincteric flow, regulated by the sphincter of Oddi which acts as a pump, is a direct measure of sphincter of Oddi function, an alteration of this trans-sphincteric flow after intragastric or i.v. ethanol may indicate Oddi dysfunction; the authors also investigated whether neural mechanisms and gastric mucosal damage might play a role in this process [25], demonstrating that both intragastric and i.v. ethanol administration altered the Oddi trans-sphincteric flow. They also suggested, in accordance with other studies [26-28], that the fall in Oddi trans-sphincteric flow might be due to the direct effects of ethanol, its metabolites (acetaldehyde) and/or other humoural agents (superoxide, endothelin-1) on sphincter of Oddi motility. Furthermore, an effect of ethanol and/or its metabolites on sphincter of Oddi nitrergic innervations was observed [25]. The authors thus concluded that reduced sphincter of Oddi function might contribute to elevated pancreatic duct pressure, which is one of the events required for the onset of acute pancreatitis [25].

There are no reports in the literature of acute pancreatitis after treatment of HCC with conventional PEI; in contrast, a case of lethal acute pancreatitis is described as a complication of intra-arterial PEI [29]. This technique can only be performed after the superselective puncture of HCC-supplying arteries, and the extreme technical difficulty of this method provides the major reason for the frequent failures of intra-arterial PEI [30].

In an interesting study on large infiltrative HCC treated with intra-arterial PEI, the volume of ethanol intra-arterially injected ranged from 12 to 50 mL [mean, 25 mL ± 13 (63% of total volume injected into tumour)] in a single session and from 0 mL to 50 mL [mean: 15 mL ± 19 (37% of total volume injected)] in the subsequent sessions [29].

A higher survival rate compared with that obtained after one-shot PEI [30] was observed with this intra-arterial PEI procedure [29]. However, the authors found that the main specific complication of this procedure, which caused the death of one of their patients, was ethanol reflux into the pancreaticoduodenal artery, a condition that can occur when the arterial branch of the HCC, in which ethanol is injected, originates from a short left hepatic artery close to the origin of the pancreaticoduodenal trunk [31].

It is obvious that in this case, the reflux of ethanol in the pancreaticoduodenal trunk was the initial cause of pancreatitis through direct induction of a toxic necrosis of the pancreas. However, we cannot rule out the possibility that also opioids may have contributed to the development of pancreatitis and that the alteration of the Oddi trans-sphincteric flow induced by ethanol may have played a role, although the authors did not mention this possibility [3].

Quite recently, we performed a "one shot" PEI (a total dose of 50 ml) into two HCC nodules of 4,6 and 3,1 cm respectively, in a patient with Child A cirrhosis. Pain management after the procedure was applied with morphine (10 mg i.v. and 10 mg s.c.), and with paravertebral block (right side) of D3-D5 by means of naropine 0,75 60 mg (total dose). On the next day, the patient developed oedematous head pancreatitis. In order to reduce his abdominal pain, treatment with opioids (morphine 8 mg/i.v. and tramadol 50 mg/i.v.) was maintained until two days after PEI; then, only tramadol 50 mg/i.v b.i.d. was continued until nine days after PEI. Despite an appropriate medical treatment of oedematous head pancreatitis and paralytic ileus (with octreotide, subcutaneous longastatine, hydration infusion and antibiotics), the patient's clinical condition further worsened and free subdiaphragmatic airways, mild abdominal fluid collection and necrosis of the head of the pancreas were observed on a contrast CT. Surgical intervention was mandatory and histological examination of the resected organs showed necrosis of the gallbladder, chronic steatophagic inflammation of the omentum, steatonecrosis of the gastric antrum with microerosive gastritis, haemorrhagic necrosis of the appendix and steatonecrosis of both the pancreatic head and the duodenum. After a few weeks, the patient fell into a hepatic coma and died of multiorgan failure and end-stage hepatic insufficiency.

Based on the data available in the literature, our opinion is that acute pancreatitis may develop in cirrhotics with HCC treated with opioids to alleviate their pain after PEI. The mechanism through which ethanol may induce pancreatitis is partially known. After PEI, ethanol cannot easily diffuse into the surrounding non-tumoural tissue, since that tissue is firmer than the tumour structure. Therefore, in this case, the development of pancreatitis may have been favoured by the ensuing treatment with opioids although it cannot be ruled out that ethanol may have played a role; in fact, possible mechanisms of ethanol-induced pancreatitis may be pancreatic duct constriction, Oddi trans-sphincteric flow alteration, metabolic effects, direct cellular toxicity, all of which have been previously discussed [22-25].

An experimental animal study on rats with BW7756 hepatoma, performed to compare efficacy and safety of two percutaneous ablation methods [PEI and PAI (percutaneous acetic acid)], showed that PEI had a lower mortality rate for complications than PAI, and that none of the complications from either procedure was due to pancreatitis [32]. In fact, autopsies revealed that the deaths of the rats were due to massive liver necrosis (about 40%) with diaphragma involvement, or to complete inferior vena cava thrombosis with extension to the right atrium.

In this experiment, PEI was performed under general anaesthesia and opioid analgesics were not administered: this might be the reason why no evidence of pancreatitis was observed [32].

It is true that pancreatitis after treatment with PEI of cirrhotics with HCC is a very rare complication, but these data, taken together, show that both opioids and ethanol may induce acute pancreatitis.

It is well established that opioids can favour the development of pancreatitis through a constriction of the Oddi's sphincter. The fact that i.v. ethanol may alter the function of the Oddi's sphincter [25] suggests that both in intra-arterial PEI and in "one shot" PEI, the pathogenesis of pancreatitis may have also been due to mechanisms of motility dysfunction of the Oddi's sphincter.

Therefore, the combined administration of ethanol and opioids may greatly favour the development of pancreatitis in both procedures.

According to Beger et al., mortality after acute pancreatitis is 7.6% when less than 30% of the pancreas is necrotic and 24% when up to 50% of the pancreas is necrotic. However, mortality is 34.3% when there are additional extrapancreatic fluid effusions [33]. According to Rau et al. and Hartwig et al., the mortality rate after acute pancreatitis varies from 20 to 30% [34,35].

In animal models of severe necrotizing pancreatitis, mortality is promoted by sepsis and by the development of a systemic inflammatory response syndrome, which, in turn, causes lethal multiorgan failure [36,37].

Therefore, given the elevated mortality rate of pancreatitis, more attention is necessary when pain is treated with opioids in cirrhotics with HCC after PEI.

## Competing interests

The authors declare that they have no competing interests.
